# Editorial: The intricate innate immune-cancer cell relationship in the context of tumor angiogenesis, immunity and microbiota: The angiogenic switch in the tumor microenvironment as a key target for immunotherapies

**DOI:** 10.3389/fimmu.2022.1045074

**Published:** 2022-10-06

**Authors:** Lorenzo Mortara, Andrew V. Benest, Lisa Derosa, Salem Chouaib, Domenico Ribatti

**Affiliations:** ^1^ Laboratory of Immunology and General Pathology, Department of Biotechnology and Life Sciences, University of Insubria, Varese, Italy; ^2^ Endothelial Quiescence Group, Centre for Cancer Sciences, Biodiscovery Institute, School of Medicine University of Nottingham, Nottingham, United Kingdom; ^3^ Institut National de la Santé et de la Recherche Médicale, U1015, Institut Gustave Roussy, Villejuif, France; ^4^ Institut National de la Santé et de la Recherche Médicale, U1186, Institut Gustave Roussy, Université Paris-Saclay, Villejuif, France; ^5^ Thumbay Research Institute of Precision Medicine, Gulf Medical University, Ajman, United Arab Emirates; ^6^ Department of Basic Medical Sciences, Neurosciences and Sensory Organs, University of Bari Medical School, Bari, Italy

**Keywords:** Cancer, tumor microenvironment, tumor angiogenesis, Immunity, microbiota, immune checkpoint inhibitors, immunotherapies

The tumor microenvironment (TME) represents a complex multicellular network which comprises host-derived stromal, immune and endothelial cells with potential dual role in tumor development and dissemination. For example, immune cells have the ability to recognize and orchestrate anti-tumor responses leading to cancer cell death, however in the meantime they can become exhausted whereas innate immune cells can acquire pro-tumorigenic and/or pro-angiogenic activities.

This Research Topic was designed to dissect various aspects of interactions that tumor cells must set up with the TME to trigger tumor blood vessel proliferation, to tamper host anti-tumor responses and to modulate microbiota, and to investigate feasibility to target these pathways to improve immunotherapies.

Here, Genova et al. examines the impact that the TME can have on immune checkpoint inhibitors (ICIs) in non-small cell lung cancer (NSCLC). They discuss on the pro-angiogenic and immunosuppressive role of the TME exerted by many distinct cells as well as on multiple clinical studies focusing on alternative immune checkpoint receptors that could lead to exhausted T and natural killer (NK) cells and resistance to ICIs. Importantly, they provide an update on novel predictors of response from currently available ICI and novel therapeutic targets. In fact, there are many promising preclinical and trials data in NSCLC, where in parallel with classical ICIs targeting PD-1/PD-L1, new target molecules could be used, such as: LAG-3 and TIM-3.

In turn, the review by Baci et al. takes under consideration the role of tumor immune microenvironment in NSCLC and the interactions between tumor cells and immune infiltrate with the aim to define new targetable drivers of immunotherapy. In particular, they pinpoint the effects exerted by neutrophils, myeloid-derived suppressor cells (MDSCs), NK cells, NKT cells, dendritic cells (DCs), Treg cells and mast cells on the orchestration of primary resistance to ICIs. This review also includes the discussion about the relevance of combination of anti-angiogenic therapies with ICIs.

Concerning anti-angiogenesis therapy, Solimando et al. in their mini-review, examine this phenomenon in metastatic castration-resistant prostate cancer (mCRPC). Targeting angiogenesis has failed to impact overall survival in patients with mCRPC despite promising preclinical and early clinical data. Narrowing the gap between the bench and bedside appears critical for developing novel therapeutic strategies. Several other compounds with known anti-angiogenic properties, including metformin or curcumin, are currently investigated. Angiogenesis-targeting strategies include biomarker-guided treatment stratification as well as combinatorial approaches. Beyond established angiogenesis inhibitors, therapies aiming at prostate specific membrane antigen (PSMA) have a substantial anti-angiogenic effect, due to PSMA´s expression in tumor vasculature.

Understanding the interactions between all the constituents of the TME remains a challenging task. Currently most patients still do not benefit from cancer immunotherapies notably because of the hostility imposed by the hypoxic microenvironment inducing immune suppression and tumor plasticity and resistance. Khouzam et al. review the mechanisms by which hypoxic stress impacts immune cell functions and how that could translate to predicting response to immunotherapy. Of particular interest is the discussion relating to how multi modal diagnostic techniques are being aligned with *in silico* approaches. Along the same line of research, Janji and Chouaib summarize the contribution of hypoxic stress to tumor progression, and its impact upon conventional anti-tumor therapies. However, although increasing evidence, the acceptance that targeting hypoxia in combination with immunotherapy might offer further clinical benefit is less well established. HIF1a signaling is a known modulator of multiple inflammatory cytokines and checkpoint expressions and therefore offers new avenues to explore as immunotherapy becomes a standard treatment.

Interestingly, Wang et al. in their review investigate the relevance of the TME in the hepatocellular carcinoma, a cancer with high worldwide incidence and with serious therapeutic implications. They illustrate the possibility of targeting the TME using immunomodulatory therapy (ICIs, new immune checkpoints, combination of ICIs with multiple kinase inhibitors), or oncolytic viruses or anti-angiogenesis therapies.

Taken together, the TME is not simply pro-angiogenic or pro/anti-inflammatory, rather is a dynamic milieu of complex interactions and cellular consequences. Of note, Xu et al. highlight the practical application of this in their report; the particularly rare splenic angiosarcoma is treatable with anti-PD-L1 antibody and tyrosine kinase receptor inhibitors. Whereas this is a case report, and full clinical trials will need to be registered and completed it offers promise to an otherwise poor prognosis, indeed at 3 months no metastatic colonization was observed. Of particular interest is the use of computed tomography (CT) and magnetic resonance imaging (MRI) to assess the efficacy of combination treatment.

Instead, Etxebeste-Mitxeltorena et al. in their review, analyze the role of adoptive cellular immunotherapy using chimeric antigen receptor (CAR)-modified T cells and NK cells in cancer. Whereas CAR-T cells induce outstanding responses in a subset of hematological malignancies, responses are much more deficient in solid tumors. Authors describe plasticity of immune cells and how these cells change their activity and phenotype depending on the stimuli they receive from molecules secreted in the TME. For example, this phenomenon could affect tumor cell phagocytosis by macrophages, which is required to remove dying tumor cells after the attack of NK cells or CAR-T cells, and it can be avoided in the TME.

Concerning ICIs resistance in solid tumors, the review by Roberto et al. analyzes how microbiota is affected by intestinal microenvironment and how microenvironment alterations may influence the response to ICIs. They showed how diet is emerging as a fundamental determinant of microbiota’s community structure and function and describe the role of certain dietary factors, as well as the use of probiotics, prebiotics, postbiotics, and antibiotics in modifying the human microbiota. Finally, they shed new light on the possibility of administering fecal microbiota transplantation to modulate the gut microbiota in cancer treatment.

Within the frame of this Research Topic, the article of Qing et al. probed the Cancer Genome Atlas (TCGA) and the GEO repository for gene signatures relating to angiogenesis and immune cells infiltration and combined the transcriptomic data with prognostic data to predict therapeutic responses. The resultant data were used to generate a prognostic nomogram, allowing clinicians to match tumor characteristics with potential personalized therapeutic opportunities.

On the other hand, Zhang et al. in their work systematically collected and evaluated the infiltration pattern of 65 immune cells. They constructed the immune cell pair (ICP) score based on the cell pair algorithm across 12 independent cancer types. The ICP score showed reliability and efficacy in predicting the survival of patients with gliomas, in pan-cancer samples, and six independent cancer types. Moreover, the ICP score was correlated with the genomic alteration features in gliomas, exhibited a remarkable association with multiple immunomodulators that could potentially mediate immune escape, and predicted immunotherapeutic responses with a high sensitivity.

## Author contributions

All authors listed have made a substantial, direct, and intellectual contribution to the work and approved it for publication.

## Conflict of interest

The authors declare that the research was conducted in the absence of any commercial or financial relationships that could be construed as a potential conflict of interest.

## Publisher’s note

All claims expressed in this article are solely those of the authors and do not necessarily represent those of their affiliated organizations, or those of the publisher, the editors and the reviewers. Any product that may be evaluated in this article, or claim that may be made by its manufacturer, is not guaranteed or endorsed by the publisher.

